# Stakeholder Involvement in the Development of a New Proactive Clinical Review of Patients Prescribed Opioid Medicines Long‐Term for Persistent Pain in Primary Care

**DOI:** 10.1111/hex.70264

**Published:** 2025-04-25

**Authors:** Sarah A. Harrisson, Clare Jinks, Nicola Cornwall, Charlotte Woodcock, Lisa Dikomitis, Toby Helliwell, Adele Higginbottom, Janet Lefroy, Roger Knaggs, Tamar Pincus, Miriam Santar, Claire Sillitto, Matthew Webb, Simon White, Christian D. Mallen, Julie Ashworth

**Affiliations:** ^1^ Centre for Musculoskeletal Health Research, Primary Care Centre Versus Arthritis School of Medicine, Keele University Keele UK; ^2^ Warwick Medical School University of Warwick Coventry UK; ^3^ Midlands Partnership University NHS Foundation Trust, Haywood Community Hospital Stoke‐on‐Trent UK; ^4^ Division of Pharmacy Practice and Policy School of Pharmacy, University of Nottingham Nottingham UK; ^5^ Pain Centre Versus Arthritis Clinical Sciences Building, City Hospital Nottingham UK; ^6^ School of Psychology, Highfield Campus University of Southampton Southampton UK; ^7^ Primary Care Research Centre University of Southampton, Aldermoor Health Centre, Aldermoor Close Southampton UK; ^8^ School of Pharmacy and Bioengineering Keele University Keele UK

**Keywords:** chronic non‐malignant pain, community of practice, complex research intervention, implementation, methodology, primary care, service user involvement

## Abstract

**Background:**

Stakeholder involvement is a core element of the Medical Research Council (MRC) framework for developing and evaluating complex interventions, but approaches to involve stakeholders are not well‐reported. We outline how stakeholders contributed to co‐designing a Proactive clinical Review of patients taking Opioid Medicines long‐term for persistent Pain led by Pharmacists working in primary care Teams (the PROMPPT intervention—a review and pharmacist training package).

**Methods:**

We brought key stakeholders together to co‐design the PROMPPT intervention using a person‐based approach, alongside evidence from best practice guidance. We established a community of practice comprising three complementary groups: a patient advisory group, a pharmacist advisory group and a mixed stakeholder group. Patient stakeholders were identified from an existing patient involvement group. Professional stakeholders were identified using networks and social media. The three groups met in iterative workshops with predefined aims. We offered reimbursement for the stakeholders' time.

**Outcomes:**

The patient advisory group (*n* = 10), pharmacist advisory group (*n* = 6) and mixed stakeholder group (*n* = 16) each met for 2 or 3 workshops between April 2019 and February 2020. Stakeholders had expertise, often cross‐cutting, in lived experience, persistent pain, opioids, delivering primary healthcare and/or promoting behaviour change. Patient stakeholders provided their perspectives of consulting about their pain and opioids. Pharmacist stakeholders provided their perspectives on how pain reviews were happening in practice and on considerations for training (e.g., vignettes and experiential learning were considered important). The mixed stakeholder group provided a breadth of views highlighting current practice, including the value of engaging the wider GP practice team, issues around clinical responsibility for prescribing and the fact that international clinical guidance was not always relevant to UK primary care.

**Conclusions:**

By understanding the context of the PROMPPT intervention, stakeholders worked to develop a new pharmacist‐led primary care review ahead of feasibility testing. We make recommendations for future developers of complex interventions.

**Patient and Public Contribution:**

Patient stakeholders, including a lay co‐applicant (C.S.) supported by a PPI support worker (A.H.), helped develop and refine the intervention. C.S. and A.H. read and contributed to the initial manuscript and approved the final manuscript.

**Abbreviations**
GP: General Practitioner
MRC: Medical Research Council
NHS: National Health Service
NIHR: National Institute of Health and Social Care
PPI: Patient and Public Involvement
PROMPPT: Proactive clinical Review of patients taking Opioid Medicines long‐term for persistent Pain led by Pharmacists in primary care Teams
UK: United Kingdom

## 1 Background

Opioid prescribing is common for people with persistent pain (pain lasting 3 months or longer and not caused by cancer) despite a lack of evidence for their long‐term effectiveness in this population and growing evidence of harm [1, 2, 3]. The implementation of opioid management guidance, such as the recommendation that patients prescribed opioids should have a regular, comprehensive review [4, 5, 6], has been limited in real‐world settings [7, 8]. In the United Kingdom, most opioid prescribing happens in primary care, where a shift to multidisciplinary working has seen an expansion in pharmacists working in GP practices [9, 10]. These practice pharmacists seem well‐placed to take a proactive role in reviewing patients prescribed regular opioids for persistent pain. However, both patients and healthcare professionals often find conversations about opioids difficult [11], and there is no evidence about what an effective pharmacist‐led primary care review for patients prescribed opioids should look like.

The Medical Research Council (MRC) framework is regarded as the gold standard for developing complex interventions, which are context‐dependent and consist of multiple components that often aim to change behaviour and improve outcomes related to a specific health or social care issue [12]. When developing new interventions, robust methodology across the whole development and evaluation pathway helps to give interventions the best chance of being effective and implemented and of being acceptable to patients receiving the intervention and the healthcare professionals delivering it [12]. There is no one‐size‐fits‐all approach to intervention development; the approach largely depends on the aim and context of the intended intervention [13]. However, stakeholder involvement is a core element of the updated MRC framework [14]. According to guidance from O'Cathain et al. [13], who outline an applied strategy for intervention developers, stakeholder involvement is considered a key action in the development of complex health interventions.

Stakeholders are defined as individuals or groups who are responsible for or affected by health‐ and healthcare‐related decisions that can be informed by research evidence [15]. Key stakeholders include patients, healthcare providers, those with responsibilities to commission and pay for healthcare services, policymakers (e.g., from professional associations) and researchers [15]. Although stakeholder involvement is recommended by major funders of research (e.g., the UK National Institute of Health and Care Research [NIHR]), many studies of intervention development have not involved stakeholders, and when they do, the methods of engagement are often not described [16].

One approach to enable meaningful involvement is to bring stakeholders together to build a community of practice [17]. A community of practice is an organised group of people with shared interests and goals, with the key functions of peer problem‐solving and generating new ideas [18]. This concept is similar to other common terms used in Patient and Public Involvement (PPI), which is defined as ‘research being carried out “with” or “by” members of the public rather than “to”, “about”, or “for” them [19]. Furthermore, the Health Research Authority in the United Kingdom defines PPI as ‘all the ways in which the research community works together with people including patients, carers, advocates, service users and members of the community’ [20, 21]. For this paper, we use the term ‘stakeholder involvement’ in this broader sense. This paper describes how we used this approach to embed stakeholder involvement into the development of a new complex intervention—a Proactive clinical Review of patients taking Opioid Medicines long‐term for persistent Pain led by Pharmacists working in primary care Teams (the PROMPPT intervention).

**Figure 1:**
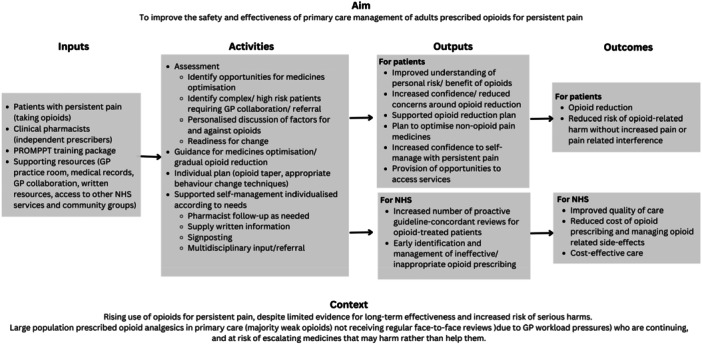
The PROMPPT Logic model.

The aim of the PROMPPT intervention (a review and a pharmacist training package) is to support patients with persistent pain in primary care to reduce opioids, where appropriate, without increasing pain or pain‐related interference. In line with the MRC Framework [12], we combined a person‐based approach [22] combined with best practice guidance, theory and stakeholder involvement work. Whereby the focus of person‐based approaches is on understanding and accommodating the perspectives of those who will use the intervention [22]. Before the award of funding, targeted stakeholder activity supported the development of the logic model (Figure 1).

**Figure 2:**
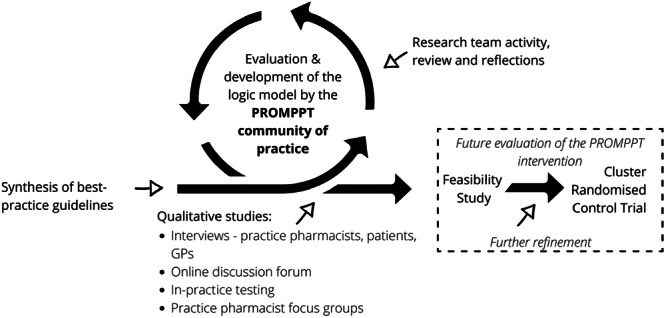
Research and stakeholder activity in the development of the PROMPPT intervention.

This paper describes the involvement work to optimise the logic model, design the PROMPPT intervention and refine it ahead of formal feasibility testing. The stakeholder activity was preceded by a synthesis of published guidance on opioid management for persistent pain and carried out alongside primary qualitative data collection (interviews, online discussion forum, in‐practice testing and focus groups) [23, 24, 25] and ahead of formal evaluation as illustrated in Figure 2.

Our work example illustrates how we established, built and maintained a community of practice to co‐design the PROMPPT intervention with stakeholders. We have used the short‐form version of the Guidance for Reporting Involvement of Patients and the Public (GRIPP2‐SF) [26] checklist for reporting PPI throughout the manuscript (see table in Supplementary Information).

## 2 Methods

We established three complementary stakeholder groups, specifically, a mixed stakeholder group and, to ensure adequate representation of the target population (those who will receive and deliver the intervention), a patient and a pharmacy advisory group. The groups met in iterative workshops, each guided by predefined aims, informed by the logic model, the findings from synthesised published guidance and emerging insights from the qualitative work [23, 24, 25]. We offered all stakeholders reimbursement for their time aligned with national guidance. Patient stakeholders' time aligned with NIHR public contributor payment policy [27]. That professional stakeholders were reimbursed commensurate with their qualifications [28]. To keep the stakeholders up to date with the progress of the research programme, we created and distributed newsletters, posted regular updates on the study website (promppt.co.uk) and used social media. Stakeholder feedback was collected and reviewed by the research team using group discussions, written notes and follow‐up communications. This aimed to gather immediate reactions, key takeaways and improvement suggestions. Decision‐making was made by the research team, with decisions communicated back to stakeholders through plain English summaries.

### 2.1 Patient Advisory Group

A group of patient stakeholders was convened with the aims of (1) providing patient perspective on the design of personalised discussions about opioids and strategies to support self‐management and (2) co‐designing patient resources. Patient stakeholders with experience of taking opioid medicines for persistent pain were recruited from the Research User Group (RUG) at the School of Medicine, Keele University. The patient stakeholders were supported by a dedicated PPI support worker (A.H.) before, during and after meetings [29]. The PPI support worker, who has personal experience of living with persistent pain, supported the day‐to‐day organisation of patient stakeholder activity. This group included co‐author C.S., who also had a link role with the mixed stakeholder group and the research team. The group, which was convened to support the funding application and continued post‐funding to support intervention development, met for face‐to‐face workshops in buildings on the university campus.

Each workshop had a similar structure, starting with a researcher‐led presentation, followed by a facilitated discussion, action planning, summary and next steps. The presentations were developed to outline the aims of the meeting, to provide some context in terms of how the research programme had moved forward towards its goals and to summarise key and complex issues [30]. Meetings were audio‐recorded, with consent, for the purposes of capturing the discussions accurately. Written materials were in plain English, and the use of jargon was minimised [31].

### 2.2 Pharmacy Advisory Group

Pharmacists with experience working in primary care teams and/or with experience working with patients prescribed opioids for long‐term persistent pain were invited to join the pharmacy advisory group. The emphasis of the pharmacy advisory group workshops was placed on seeking the experiences and advice from members of the group with the aim of (1) providing a pharmacist perspective on the design of personalised discussions about opioids and (2) co‐designing the pharmacist training package. Pharmacists were recruited from across the United Kingdom by advertising through professional networks and using social media. Meetings were held remotely (using teleconference services) and were audio‐recorded with consent. To take into account scheduling difficulties, multiple workshops were held, with a minimum of two pharmacists per meeting. Workshops were facilitated by a member of the research team who was an academic pharmacist (S.W.). Documents outlining the background and aims of each of the workshops were circulated in advance of the meetings.

### 2.3 Mixed Stakeholder Group

The mixed stakeholder group was convened to (1) review and prioritise techniques and strategies considered best practice in persistent pain management (e.g., regarding prescribing, opioid reduction and supported self‐management) identified in the synthesis of best practice guidelines and (2) agree on the content and structure of the PROMPPT review ahead of formal feasibility testing.

The mixed stakeholder group comprised patient stakeholders with relevant lived experience and healthcare professionals, with expertise regarding opioids, persistent pain and/or delivering primary care services. Healthcare professional stakeholders were recruited through professional networks. Patients with experience working alongside healthcare professionals (e.g., in project steering committees) were recruited from the RUG. Patient stakeholders were accompanied by the PPI support worker during the workshop and, afterwards, attended a debriefing session. Before the award of funding, patient stakeholders themselves shared that mixed groups can often feel intimidating, and they suggested including patients with prior experience working alongside healthcare professionals (e.g., in project steering committees).

The mixed stakeholders convened for face‐to‐face workshops. The location, structure of the workshops and format of written information were as per the patient advisory groups. If stakeholders were not able to attend, individual meetings were scheduled. To support critical reflection and disseminate findings from these workshops to the patient advisory group, the facilitating researchers subsequently met with patient stakeholders, co‐author C.S. and the PPI support worker. Parallel sessions were held with a pharmacist stakeholder with the same purpose.

### 2.4 Outcomes

#### 2.4.1 Characteristics of Community of Practice Members

##### 2.4.1.1 Patient Advisory Group

In the patient advisory group (*n* = 10), all members had experience with opioids, either personally or by caring for others who had taken opioids regularly for persistent pain. Previous experience of involvement in research varied, and three members of the group had no previous involvement. Most of the patient stakeholders were female (*n* = 8), and they all came from the local area (Staffordshire, the United Kingdom).

##### 2.4.1.2 Pharmacy Advisory Group

The Pharmacy advisory group comprised 6 pharmacists with cross‐cutting experience working in GP practices, community pharmacy, pain management and/or commissioning. The Pharmacy stakeholders expressed a wide breadth of professional interests in pharmacological and non‐pharmacological pain management, decreasing dependence forming medicines, medicines optimisation, pharmacist education, research, non‐medical prescribing and/or having advised the national clinical practice of pain management.

##### 2.4.1.3 Mixed Stakeholder Group

The mixed stakeholder group (*n* = 19) comprised of two patient contributors, two GPs, one practice nurse, two pharmacists, two practice managers, one social prescriber, two physiotherapists and one clinical psychologist from a community pain service, one psychological therapist from a community mental health team, two psychiatrists from addiction services and one academic health psychologist. One of the GPs and one of the pharmacists had responsibility for the commissioning of primary care services.

#### 2.4.2 Stakeholder Contributions to Co‐Design and Optimisation of the PROMPPT Review

The stakeholder involvement work comprised seven workshops between April 2019 and January 2020 (mixed stakeholder group *n* = 3, patient advisory group *n* = 2 and pharmacy advisory group *n* = 2). The views and recommendations of stakeholders and the actions taken as a result are presented in Table 1.

**Table 1:** Work undertaken by stakeholders to co‐design the PROMPPT intervention.

Date and group	Discussion topics	Views and recommendations by the stakeholders for the proposed PROMPPT review	Actions taken by the research team
11 April 2019 Mixed Stakeholder Group	Current clinical guidance on opioid reduction for patients with persistent pain	Care of patients should be individualised according to patient needs Not all evidence (including patient resources) relevant to UK primary care	PROMPPT designed as a personalised review Acknowledged and collated existing patient and training resources that could inform PROMPPT review
	Potential facilitators and barriers to pharmacists in GP practices proactively reviewing patients on opioid medicines	GP practice managers, practice nurses and social prescribers are important stakeholders In the context of persistent pain and opioid reduction, qualitative studies should explore engagement and involvement of the wider GP practice team and other services, shared decision‐making, patient and pharmacist concerns and clinical responsibility for changes to prescribing	Nurses, GP practice managers and social prescribers were invited to join the mixed stakeholder group. All stakeholder recommendations were included in revised topic guides for interviews and pharmacist focus groups
23 and 25 July 2019 Pharmacist Advisory Group	Overarching principles of the review and the training package	A holistic approach is important Vignettes, real‐life experiential learning and mentor support recommended in the training	Developed strategies to facilitate a holistic review (e.g., patient resources and key skills for pharmacists) Simulated consultations were incorporated into the training
	Assessing readiness to reduce opioids	A framework may be useful to support pharmacists in identifying how ready patients are	Developed guidance on assessing readiness to change into training
4 September 2019 Patient Advisory Group	The approach of the proposed intervention	The review and patient resources should focus equally on living well with pain and reduction in opioids	Pharmacist training package to include having conversations about living well with pain as well as reducing opioids
	Discussed options for patient resources	Patient resources should be available in different formats (printable, e‐version and video) Letters inviting patients to a PROMPPT review may be more credible if sent from the GP practice Embed hints, tips and stories into resources	Early versions of patient resources developed with the patient group and then discussed with the mixed stakeholder group (19 September 2019) Considered options for including GP letterheads on invitation letters A video sharing the story of a person with relevant lived experience of reducing opioids was created
19 September 2019 Mixed Stakeholder Group	Communicating with patients outside of the PROMPPT review	Consider using text messaging; contact between appointments may not be feasible; take account of administrative time	Considered options for using text messages within primary care electronic systems; setting realistic expectations for contact between appointments Discussed contact between appointments with the patient advisory group (2 October 2019)
	Referrals and signposting	The intervention may facilitate onward referrals to Physiotherapy and social prescribing There is often variation in commissioned services, which may impact onward referrals	Checked and confirmed that the topic of referrals and the impact of variation in commissioning was scheduled to be discussed in focus groups
	Current roles and responsibilities of pharmacists working in GP practices	Roles, responsibilities and previous experience of pharmacists vary and may impact on training needs NHS plan for pharmacists is evolving—the PROMPPT review will need to be future‐proof	Considered different approaches to training (e.g., tailored specifically to individual needs vs. a comprehensive approach for all) Checked and confirmed that the topic of pharmacy roles and training needs was scheduled to be discussed in focus groups
	A shared decision‐making tool to help pharmacists have conversations about opioids	Shared decision‐making tools can be useful and could be a conversation aid There are Pro's and con's of developing a hi‐tech vs. lo‐tech tool (different tools for different people)	Explored the feasibility of developing a bespoke shared decision‐making tool A prototype shared decision‐making tool was developed and discussed with the patient advisory group (31 October 19)
	Early versions of patient resources to support the PROMPPT review (‘Pain Concerns Form’ and ‘Patient Invitation letter’)	To facilitate patient engagement, getting the language right in the documents is important in patient resources—some changes recommended to both the Pain Concerns Form and the Patient Invitation Letter	Reviewed and amended text and format of resources with a focus on using non‐judgmental language New iterations were reviewed by members of the patient advisory group (2 October 2019)
2 October 2019 Patient Stakeholder parallel session	Pain Concerns Form and Patient Invitation Letter	Patient resources should be personalised where possible, and the text should be appealing	The Pain Concerns Form and Patient Invitation Letter were simplified to improve readability
	Communicating with patients outside of the PROMPPT review	A clear mechanism about how and when to get in contact with the pharmacist is helpful to patients if they are having a setback, but don't often use it	Setback planning to be discussed with the pharmacy advisory group (16 and 31 October 2019)
31 Oct 2019 Patient Advisory Group	Shared decision‐making tool	The tool may move the pharmacist away from a holistic conversation	Discussed the utility of progressing with the shared decision‐making tool, given that it may not be deemed a useful addition to the consultation.
	Patient resources	Agreement that the identified patient resources seem to be from ‘trustworthy’ sources and comprehensive. Some changes to the text may be beneficial	Final refinements made to the prototype patient resources ready for in‐practice testing
16 and 31 October 2019 Pharmacist Advisory Group	The PROMPPT review	When planning opioid reduction, it can be helpful to discuss potential setbacks (e.g., an increase in pain) ahead of an agreement to make a reduction	Added content on setback planning to the pharmacist training for the feasibility study
	Collaborating with GPs	Deterioration in mental health, rapid increase in reported pain and other new symptoms are important reasons for pharmacists to seek help	Added content on when to collaborate with the GP Added instructions to patient resources on how, if needed, patients should contact the pharmacist between appointments
30 January 2020 Mixed Stakeholder Group	The PROMPPT review	Asking the patients to opt‐in to attend a review may identify those ready to engage in an opioid reduction Consider whether the aims of the PROMPPT review are achievable in 30 min Embed all research‐specific documents into existing primary care systems. Avoid duplication	Considered and implemented an opt‐in approach Time taken to conduct the review and to complete associated documentation to be recorded and assessed in the feasibility study Study documentation embedded into GP clinical systems
	The pharmacist training package	Ongoing mentoring beyond the training package was important (e.g., peer‐to‐peer and in‐practice support), especially given the emerging roles and different levels of clinical experience Online training could offer pharmacists and GP practices important flexibility and scale‐up	Developed the role of the clinical champion to mentor pharmacists delivering PROMPPT Ahead of the feasibility study, recruited pharmacists with relevant experience to the role Amended the feasibility study protocol to allow for online training
	Engaging the wider GP practice in PROMPPT	Consider engaging clinical and non‐clinical staff to raise awareness of the practice's involvement in delivering PROMPPT	Developed brief training and information for all staff
12 February 2020 Pharmacist Stakeholder parallel session	Existing models of pharmacist training	There are existing online training courses for pharmacists but little cross‐over with the aims of PROMPPT. Important for pharmacists to reflect and determine the impact of training	A meeting of researchers with experience in developing online training considered barriers and facilitators to future delivery/implementation of the PROMPPT training Incorporated reflective practice into training
13 February 2020 Patient Stakeholder parallel session	Self‐care for pain self‐management	It is important but difficult for patients to self‐care; pharmacists need to ‘sell’ this approach Pharmacists could need support as sometimes conversations can be difficult (own self‐care)	Refined aims of training to have conversations about self‐care alongside opioid reduction Content on pharmacist self‐care added to the training

All three stakeholder groups started by discussing the overall principles for the new PROMPPT intervention and then moved towards discussing specific elements of the logic model. In the early stages, the views and recommendations of the stakeholders often fed directly into primary qualitative data collection (e.g., informing topic guides for the interviews). Other recommendations were carried forward for further discussion in later workshops (e.g., to what extent the intervention should focus on pain self‐management). Towards the end of the stakeholder consultation phase, recommendations (e.g., that online training would offer important flexibility and potential for scale‐up) were fed directly into the PROMPPT intervention that was evaluated in the feasibility study. There were topics where there was uncertainty amongst the stakeholders, and they did not always have clear recommendations (e.g., whether the review would result in a change in onward referrals and how to manage this). In these instances, the action taken by the research team was to check and confirm that the topic of referrals was scheduled to be discussed in the qualitative studies.

Patient stakeholders provided their views on consulting with healthcare professionals about opioids and non‐pharmacological pain management strategies. They represented the views of other groups of people (e.g., elderly people and those taking high‐dose opioids). Pharmacist stakeholders provided their views from the perspective of their profession (e.g., advising on how pain reviews were happening in current practice). The mixed stakeholder group provided their views relating to current GP practice (e.g., by highlighting the value in engaging the wider GP practice team), policy (e.g., identifying issues around who takes clinical responsibility for prescribing decisions), organisations (e.g., highlighting that different areas commission different services) and cultural factors (e.g., that international clinical guidance from outside the United Kingdom was not always relevant to UK primary care).

## 3 Discussion

Best practice guidance for the development of complex interventions highlights stakeholder involvement throughout the development process as a key action for intervention developers to consider [13, 14], but this is often under‐reported. This paper provides detailed insight into stakeholder involvement in the design of a prototype PROMPPT intervention for in‐practice testing and subsequent refinement of the review ahead of a formal feasibility study. Our worked example links stakeholder involvement to each of the key actions in guidance for intervention developers [13], thus extending current thinking in this field.

Within a 12‐month period, with a clear purpose of co‐designing the new PROMPPT intervention, the PROMPPT community of practice comprised and involved many stakeholders, including a wide range of professionals. We set an ambitious agenda to incorporate the perspective of stakeholders and to learn from their experiences. We placed importance on collaboration and ongoing dialogues. At each stage, we collected evidence (e.g., emerging evidence from primary qualitative data collection) and took that to discuss with the stakeholders. We brought together a large team, the community of practice, where iterative group work and the interactions between the groups were of added value. A strength of our involvement work was the utility of the reflective approach, which helped us to better understand the views of the stakeholders and potentially helped us to make better decisions [32]; for example, on the back of concerns from the patient advisory group about acceptability, we stopped work on a prototype shared decision‐making tool. The impact of our work is that we have been able to outline recommendations, linked to the key actions [13], for researchers when planning stakeholder involvement in complex intervention development work (see Table 2).

**Table 2:** PROMPPT stakeholder involvement and recommendations for future work mapped to key actions for intervention developers (O'Cathain et al. 2019).

Key action	PROMPPT stakeholder involvement	Recommendations for optimising stakeholder involvement in the future
Plan the process	In the early stages, patient and pharmacy advisory groups informed and helped refine the research question and the protocol for the funding applications	Develop a plan to build and maintain stakeholder involvement throughout the intervention development phase, as this has implications for budget and timeline
Involve stakeholders	We reached, engaged and involved relevant stakeholders in the community of practice	To ensure the relevance of the intervention, the diversity of stakeholders should be considered. Invite stakeholders to consider who should be involved and represented
Bring together a team	Using an iterative approach, we brought together three complementary groups, each with a clear purpose to co‐design the new intervention Link roles between groups and sustained involvement over the intervention development phase provided continuity	In the early stages, discuss with stakeholders expectations for their involvement, including duration and extended roles (e.g., lay co‐applicant)
Review research evidence	Stakeholders reflected on the relevance of guidelines on opioid tapering in patients with persistent pain and on emerging evidence from primary data collection	When presenting scientific evidence, consider using creative methods to convey complex messages so that they are accessible and inclusive
Draw on existing theory	Stakeholders provided their perspective on primary data collection that was informed by theory	Having involved stakeholders to provide their perspective on the context of the intervention, consider whether the chosen theories continue to be appropriate
Articulate programme theory	Stakeholders discussed and refined elements of the logic model	In the early stages, define the aim of the involvement work, including aspects that are amenable to change and those that are not
Undertake primary data collection	Stakeholders (mostly the Patient Advisory Group) advised on elements of the qualitative studies	Whilst developing a plan, consider stakeholder involvement in parallel work to collect primary data in addition to intervention development work
Understand context	Stakeholders provided perspective based on cross‐cutting expertise and experiences	In the early stages, familiarise the stakeholders with their key role to provide perspective on the context for intervention development and this being key to future implementation
Pay attention to future implementation	Stakeholders advised on the potential for the proposed review and training to be scalable and implementable (e,g online training was considered more flexible, which would be an advantage)	
Design and refine the intervention	Stakeholders: (i) advised on barriers to and facilitators of elements of the review (e.g., a prototype shared decision‐making tool) and (ii) designed and refined patient resources incorporating patient experience	Familiarise the stakeholders and the aim of their involvement to refine elements of the logic model ahead of evaluation

We were clear from the outset that the research programme aimed to evaluate the new intervention and that it needed to be implementable in real‐world NHS settings. We worked together with stakeholders to understand the context, generate ideas (such as the importance of a holistic approach) and gain insights into the practical aspects of delivering reviews. They anticipated barriers, like the feasibility of direct phone calls to specific staff members in primary care, and identified facilitators for future implementation, such as integrating the lived experience into patient resources, which included issues around potential scale‐up (e.g., the flexibility of including online training). We have demonstrated that the stakeholders' views and recommendations identified a breadth of features of context, including those relating to the individual, profession, culture, service and organisation [33].

Identifying where there were gaps in the stakeholder's understanding was as important as taking on their views and recommendations. The gaps often reflected wider uncertainty about issues, for example, because the role of the pharmacist in GP practices was an emerging one. In these cases, the strength of our work was that the stakeholder involvement ran alongside and subsequently informed the qualitative data collection and analysis. The effectiveness of complex interventions like PROMPPT is inextricably linked to implementation and context [34]. By engaging stakeholders through iterative workshops, the co‐design approach allowed us to identify practical barriers and facilitators within the primary care setting. For instance, stakeholders highlighted organisational challenges and cultural factors that shaped the feasibility of integrating pharmacist‐led reviews, enabling us to develop the intervention aligned with real‐world contexts. Making sense of the complexity of the context in which the PROMPPT intervention was intended to be evaluated and implemented should give it the best chance of being successful in practice [33].

We deliberately set out to co‐design the new intervention, which was appropriate given the emerging role of pharmacists in primary care and the practical constraints of our research environment. The work was planned before the onset of the global pandemic in 2020. It is likely that the things we did (e.g., involving stakeholders in the initial stages of PROMPPT, the role of the PPI support worker and the stakeholder debrief sessions) helped to foster a collaborative approach [19] whilst acknowledging that the decisions were made by the research team. The role and lived experience of the PPI support worker was key to promoting trust and good working relationships with patient stakeholders and has been previously advocated in settings with under‐served populations [29]. The Covid‐19 pandemic brought about a rapid change in how research was conducted and implemented. Having spent time building and maintaining relationships and having already identified that online training could be important to consider, the intervention was quickly modified for remote delivery, given the need for social distancing.

There are some limitations to our work to consider. Whilst we involved many stakeholders representing people with different backgrounds, it is likely that some were not represented. The majority of our patient stakeholders, despite living with persistent pain, were still able to engage in valued activity such as participating in public involvement work, and we acknowledge that this is often not the case for many people from this patient population.

Most of the professional stakeholders completed their undergraduate training and gained most of their clinical experience within the United Kingdom; they were mostly in the mid‐ to late‐career stage. In the challenging context of the NHS, where there is a high turnover of staff and the proportion of the workforce moving from outside the United Kingdom is growing [35], we recognise that the voices of professionals who were either in the early stages of their career and/or were trained from outside the United Kingdom were not well‐represented. Furthermore, our approach to reimbursing professional stakeholders whilst aligned with national guidance [28] was not fully aligned with principles of equity and reciprocity. In the future, intervention developers should plan approaches, including equitable reimbursement practices, to effectively reach, engage and involve diverse patient and professional stakeholders.

At the time of our involvement work, we did not record the ethnicity of stakeholders, and our observations were that our community was limited in ethnic diversity. It is now widely acknowledged that the ethnic diversity of stakeholders and those who participate in research often does not reflect that of the wider population [36]. Additionally, the majority of patient and professional stakeholders were female, which reflects the disproportionate burden of persistent pain on women [37] but also underscores the need to address sex‐ and gender‐based differences in stakeholder involvement. Women with persistent pain are often referred for mental health support and psychological interventions, potentially reflecting a tendency to attribute their symptoms to psychological factors [38]. In contrast, men are more likely to be referred to physical health services and undergo investigations for potential underlying biological causes [38], highlighting disparities in service delivery and outcomes. Existing clinical guidance for persistent pain overlooks these differences [39]. In the future, approaches should prioritise gender responsiveness alongside ethnic diversity to better represent and address the needs of diverse populations [40]. Since the completion of our work, the National Institute for Health and Care Research has taken action to improve the representation of under‐served groups in research through public partnership [40], the development of guidance for researchers and funders [41] and the development and testing of a race equality framework [40, 42]. In the future, improving the diversity of all stakeholders and participants in research is vital [36, 43].

Both co‐design and co‐production fall under the umbrella term of ‘co‐creation’ but with nuanced differences. Whereas co‐design emphasises collaboration and consultation, co‐production involves stakeholders as equal partners with shared decision‐making authority [44, 45]. The principles of co‐production represent an aspirational goal; co‐design offers a valuable approach for researchers operating within restraints such as time and funding to develop complex interventions [46]. We hope to guide others in navigating the balance between inclusivity and feasibility. Future work should explore ways to integrate co‐production principles more fully, including engaging groups with under‐served characteristics and fostering equal decision‐making roles. We recommend that researchers and funders allocate sufficient time and resources to support such efforts.

## 4 Conclusion

We convened three complementary groups of stakeholders and brought them together in a community of practice approach to develop the PROMPPT intervention that is ready for testing in a feasibility study, ahead of a full‐scale cluster randomised controlled trial. The community was greater than the sum of its parts. Stakeholder involvement was key to understanding the breadth and complexity of the context of the new intervention. We have provided evidence that stakeholder involvement extends to all of the key actions for researchers when developing complex interventions. Bringing together stakeholders alongside theory and research evidence provides a rigorous co‐design framework within which to operationalise intervention development. Specific to stakeholder involvement, we make our own recommendations for researchers when planning complex interventions.

## Author Contributions

**Sarah A. Harrisson:** conceptualisation, investigation, writing – original draft, writing – review and editing, data curation, visualisation, methodology, project administration, resources. **Clare Jinks:** conceptualisation, investigation, funding acquisition, writing – original draft, writing – review and editing, supervision, methodology. **Nicola Cornwall:** investigation, writing – review and editing, conceptualisation, project administration, visualisation. **Charlotte Woodcock:** conceptualisation, investigation, writing – review and editing, project administration, visualisation. **Lisa Dikomitis:** writing – review and editing, funding acquisition. **Toby Helliwell:** investigation, writing – review and editing. **Adele Higginbottom:** investigation, project administration, writing – review and editing. **Janet Lefroy:** investigation, writing – review and editing. **Roger Knaggs:** investigation, funding acquisition, writing – review and editing. **Tamar Pincus:** funding acquisition, writing – review and editing, investigation. **Miriam Santar:** investigation, funding acquisition, writing – review and editing. **Claire Sillitto:** investigation, funding acquisition, writing – review and editing. **Matthew Webb:** investigation, writing – review and editing. **Simon White:** investigation, writing – review and editing, funding acquisition. **Christian D. Mallen:** investigation, funding acquisition, writing – review and editing, supervision. **Julie Ashworth:** investigation, conceptualisation, funding acquisition, writing – review and editing, visualisation, supervision.

## Conflicts of Interest

Some members of the research team have roles with and/or other current grant funding from the National Institute for Health and Care Research (NIHR). C.D.M., M.S. and L.D. are NIHR Senior Investigators, and C.D.M. is Director of the NIHR School for Primary Care Research. C.J. is a steering committee member of the NIHR Incubator for Applied Health and Care Methodology. C.J. and S.W. are members of the NIHR funding panel. S.A.H. and C.W. are NIHR Research Support Service advisors. S.A.H., C.J., C.W., L.D., T.H., R.K., T.P., M.S., S.W., C.D.M. and J.A. have active and/or completed research awards from NIHR. R.K. is currently President, and S.A.H. is an elected council member of the British Pain Society. R.K. is a member of the UK Government Advisory Council on the Misuse of Drugs. Whilst we do not believe that the roles/funding mentioned above will present a conflict of interest, we acknowledge that they could potentially be perceived as such. All other authors declare no conflicts of interest.

## Supporting information

supmat.

## Data Availability

The data supporting the findings of this study are available from the corresponding author upon reasonable request.
