# Supplementary material for: Stakeholder Involvement in the Development of a New Proactive Clinical Review of Patients Prescribed Opioid Medicines Long‐Term for Persistent Pain in Primary Care
Source: Health Expect. 2025 Apr 25;28(3):e70264. doi: 10.1111/hex.70264 (PMC12031880; doi:10.1111/hex.70264)
Supplement: Supplementary file 1 — supmat. [file HEX-28-e70264-s001.docx]

Supplementary files

**Supplementary Information to the manuscript: Stakeholder involvement in the development of a new proactive clinical review of patients prescribed opioid medicines long-term for persistent pain in primary care**

**Table.** Reporting patient and public involvement in the development of the PROMPPT review using the short form version of the Guidance for Reporting Involvement of Patients and the Public checklist (GRIPP2)*

| **GRIPP-2 Short form item** | **Description** |
| --- | --- |
| **Aims: Report the aim of Patient and Public Involvement in the study** | Based on the elements in an existing logic model, patient stakeholders met to co-design a prototype version of a proactive review carried out by pharmacists working in GP practices and a pharmacist training package (the PROMPPT review) alongside other key stakeholders and research evidence |
| **Methods: Provide a clear description of the methods used for Patient and Public Involvement in the study** | Patient stakeholders were identified from an existing research user group within the University leading the programme of research. With stakeholders from relevant health and social care professions, patient stakeholders joined a community of practice and met at regular intervals either in a mixed group or in a dedicated patient advisory group. One patient stakeholder was allocated the role of lay co-applicant and supported the development of the funding application and of the proposed review.  Financial resources were allocated to pay for their’ time, catering, and support from Patient and Public Involvement coordinator. Demographic information (including ethnicity) were not formally recorded. |
| **Study result outcomes: Report the results of Patient and Public Involvement in the study, including both positive and negative outcomes** | Patient stakeholders emphasized that the review and patient resources should focus equally on reducing opioids and supported self-management of pain. They advocated for other patients in the target population, highlighting that patient resources should be available in different formats, but should be relevant and appealing to all. They advised when they had concerns that elements of the proposed review may move the pharmacist away from a holistic conversation. |
| **Discussion and conclusion outcomes: Comment on the extent to which Patient and Public Involvement influenced the study overall. Describe positive and negative effects** | Patient stakeholders provided context from their perspective on pain management strategies, and how these may differ in sub-groups such as elderly people and those taking high dose opioids. They had a key function to inform the topics discussed as part of primary data collection and to generate ideas for patient resources. |
| **Reflections/critical perspective: Comment critically on the study, reflecting on the things that went well and those that did not, so others can learn from this experience** | On reflection, our patient stakeholders reflected some but not all of the target population and in the future, greater attempts to represent under-served and under-represented populations will be important. |

* Staniszewska S, Brett J, Simera I, Seers K, Mockford C, Goodlad S et al. GRIPP2 reporting checklists: tools to improve reporting of patient and public involvement in research. BMJ. 2017;358:j3453. Published 2017 Aug 2. doi:10.1136/bmj.j3453
